# Research on Acute Toxicity and the Behavioral Effects of Methanolic Extract from Psilocybin Mushrooms and Psilocin in Mice

**DOI:** 10.3390/toxins7041018

**Published:** 2015-03-27

**Authors:** Olga Zhuk, Izabela Jasicka-Misiak, Anna Poliwoda, Anastasia Kazakova, Vladlena V. Godovan, Marek Halama, Piotr P. Wieczorek

**Affiliations:** 1 Department of Biotechnology and Molecular Biology, Opole University, 45-040 Opole, Poland; E-Mail: olga_zhuk@uni.opole.pl; 2 Faculty of Chemistry, Opole University, 45-040 Opole, Poland; E-Mails: anna.poliwoda@uni.opole.pl (A.P.); piotr.wieczorek@uni.opole.pl (P.P.W.); 3 Department of General and Clinical Pharmacology, Odessa National Medical University, 65000 Odessa, Ukraine; E-Mails: ignozy@gmail.com (A.K.); godovan@mail.ru (V.V.G.); 4 Museum of Natural History, University of Wrocław, 50-335 Wrocław, Poland; E-Mail: marhal@uni.wroc.biol.pl

**Keywords:** psilocybin mushrooms, psilocin, acute toxicity, *head-twitch* response (HTR)

## Abstract

The pharmacological activities and acute toxicity of the psilocin (PC) and dried residues of the crude extracts of psychotropic mushrooms were investigated in mice. The hallucinogenic substances were effectively isolated, by using methanol, from the species of *Psilocybe semilanceata* and *Pholiotina cyanopus*, that were collected in the north-east region of Poland. The chemical analysis of these extracts, which was performed by liquid chromatography with mass spectrometry detection (LC-MS), indicated the presence of psilocin and other hallucinogenic substances, including indolealkylamines and their phosphorylated analogues. When the pure psilocin or fungal extracts were used, slight differences in determined LD_50_ values were observed. However, the application of PC evoked the highest level of toxicity (293.07 mg/kg) compared to the activity of extracts from *Ph. cyanopus* and *P. semilanceata*, where the level of LD_50_ was 316.87 mg/kg and 324.37 mg/kg, respectively. Furthermore, the behavioral test, which considered the *head-twitching* response (HTR), was used to assess the effects of the studied psychotropic factors on the serotonergic system. Both, the fungal extracts and psilocin evoked characteristic serotoninergic effects depending on the dose administered to mice, acting as an agonist/partial agonist on the serotonergic system. A dose of 200 mg/kg 5-hydroxytryptophan (5-HTP) induced spontaneous *head-twitching* in mice (100% effect), as a result of the formation of 5-hydroxytryptamine (5-HT) in the brain. Compared to the activity of 5-HTP, the intraperitoneal administration of 1mg/kg of psilocin or hallucinogenic extracts of studied mushrooms (*Ph. cyanopus* and *P. semilanceata*) reduced the number of *head-twitch* responses of about 46% and 30%, respectively. In contrast, the administration of PC exhibited a reduction of about 60% in HTR numbers.

## 1. Introduction

One of the sources of psychotropic substances is hallucinogenic mushrooms. The research carried out on the determination of chemical content of psychedelic mushroom species such as *Galerina*, *Gymnopilus*, *Inocybe*, *Panaeolus*, *Pholiotina* and *Psilocybe* showed that they mainly contained psilocybin and its dephosphorylated metabolite—psilocin—as well as the minor amounts of other indoleamine derivatives such as baeocystin, norbaeocystin and aeruginascin, may also have occurred [1,2,3,4,5,6,7,8,9,10,11,12]. However, in the case of aeruginascin, the pharmacology and toxicology effect has not yet been examined. 

Generally, hallucinogens cause extensive changes in the process of perception, thinking and mood of a human being, as well as depersonalisation [13]. Structurally, these substances mainly belong to two chemical classes: indoleamines and phenylalkylamines. The first one includes hallucinogens such as psilocin (PC), lysergic acid (LSD), *N*,*N*-dimethyltryptamine (DMT) and 5-methoxy-DMT (5-MeO-DMT). Studies, described in the literature, have demonstrated that these substances behave as non-specific serotonin receptor (5-HT) agonists (partial agonists), that bind with 5-HT_1A_, 5-HT_2A_ and 5-HT_2C_ receptors with various degrees of affinity [14,15]. There are many studies, performed both on animals and humans, that have proved the point that the characteristic hallucinogen effects took place as a result of an interaction of hallucinogenic substances with the 5-HT_2A_ receptor [16,17,18,19]. Such central nervous system (CNS) stimulants acting as partial agonists seem to be an attractive strategy for the discovery of new psychopharmacological agents with therapeutic advantages. 

One of the ways to study the pharmacology of receptor activity, *in vivo* assays, is the exhibition of *head-twitch* response in laboratory animals (rodents, mice). There is some evidence that HTR takes place by means of activation of the 5-HT_2A_ receptor [20,21,22,23]. Therefore, the main aim of this work was to investigate the acute toxicity and pharmacological effects of selected hallucinogenic factors in the population of mice. The group of tested hallucinogens consisted of pure psilocin and the dried residues of two crude methanolic extracts isolated from psychotropic mushrooms growing in Poland (*Pholiotina cyanopus* and *Psylocybe semilanceata*).

## 2. Results and Discussion

### 2.1. Acute Toxicity

**Figure 1:**
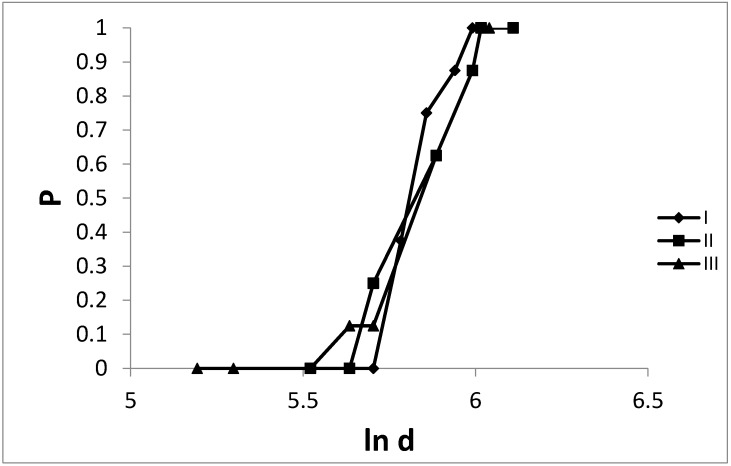
The probability (P) of the lethal effect in the population of the tested mice after a single intraperitoneal administration of the mushroom extracts (I—*Ph. cyanopus*, II—*P. semilanceata*) at dose 200–450 and psilocin (III) in the doses from 180–420 mg/kg (ln d).

During the evaluation of acute toxicity, in the course of a 24-h experiment, visible changes in the behavior of the animals under investigation were observed. In the case where the application of doses was above 200 mg/kg, the disturbance of the spontaneous conditional reflex and activity maintenance (hyperactivity after the administration of the preparation, that decreased later) was observed. The probability (P) of lethal effect (acute toxicity) as a result of the examined extracts (I–II) and psilocin (III) are presented in Figure 1. 

As it can be seen, the range of the administrated doses resulting in lethal effect was narrow—no toxic effect was observed for doses of extract I from 280 to 300 mg/kg, from 250 to 280 mg/kg for extract II and respectively 180–250 mg/kg when pure psilocin was used. Higher amounts (400 and 410 mg/kg) lead to a lethal effect of 100%. Furthermore, the LD_50_ values calculated by means of the Kerber’s method for the examined hallucinogenic factors were determined. The obtained data are shown in Table 1. 

**Table 1:** LD_50_ values (mg/kg) (± SD, *n* = 10) of the lethal effect in mice for studied methanol extracts and psilocin.

LD_50_ values		
methanol extract I *Pholiotina cyanopus*	methanol extract II *Psilocybe semilanceata*	Psilocin
316.87 ± 1.63	324.37 ± 2.04	293.07 ± 1.02

The comparison of the obtained LD_50_ values for synthetic psilocin and fungal extracts in a Student’s t-test showed insignificant differences, (*p* < 0.001). However, the application of pure psilocin exhibited the highest toxicity effect in the comparison to the mixture of the used methanolic extracts. No effects were observed in the control group where all individuals remained alive. 

The chemical analysis of the content of tested methanolic mushroom extracts, performed by liquid chromatography with mass spectrometry detection, indicated the presence of various indolealkylamines derivatives and their phosphorylated analogues (Table 2). 

**Table 2:** Indolealkylamin hallucinogens determined in methanolic extracts from *Pholiotina cyanopus* and *Psilocybe semilanceata* by liquid chromatography with mass spectrometry detection (LC-MS). ND—not detected.

Determined compounds	Chemical formula	Percentage content in dry mass of mushroom		Percentage content in dry extract	
		*Ph. cyanopus*	*P. semilanceata*	*Ph. cyanopus*	*P. semilanceata*
Norbaeoystin	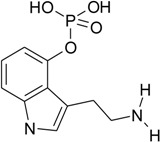 C_10_H_13_N_2_O_4_P	0.053 ± 0.004	0.077 ± 0.006	0.424 ± 0.032	0.481 ± 0.038
Baeocystin	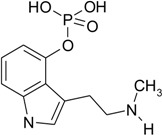 C_11_H_15_N_2_O_4_P	0.16 ± 0.01	ND	1.28 ± 0.08	ND
Psilocybin	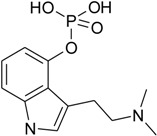 C_12_H_17_N_2_O_4_P	0.9 ± 0.08	1.46 ± 0.12	7.2 ± 0.64	9.125 ± 0.75
Psilocin	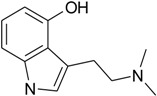 C_12_H_16_N_2_O	0.17 ± 0.01	0.24 ± 0.02	1.36 ± 0.08	1.5 ± 0.125
Aeruginascin	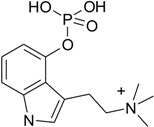 C_13_H_20_N_2_O_4_P	0.011 ± 0.0007	0.022 ± 0.01	0.88 ± 0.006	0.138 ± 0.062

The analyzed extracts contained a significant concentration level of psilocybin, the phosphorylated form of psilocin. The content of PB was about 5–6 times higher than PC. Furthermore, both PB and PC occurred in the largest amount in the tested extracts, compared to other hallucinogens. Their percentage content was over 1% of the weight of the dry mushrooms (over 8% of dry extracts), whereas the rest of the determined indole compounds (like baeocystin, norbaeocystin and/or aeruginascin) showed a level below 0.05% (the content below 0.35% of dry extracts). Taking into account the obtained results of acute toxicity and designated LD_50_ values it can be stated, that synergistic effect of indole compounds presented in the methanolic extracts was observed. It should be underlined that the observed toxic effect of mushroom extracts is much stronger than if the pure psilocin was used. In addition, it is well known that psilocybin, when administrated into living organisms, is metabolized to psilocin [24]. Therefore, it seems that the actual toxic effect is mainly responsible for the psilocin that correlated with the experiments where LD_50_ values were evaluated. Furthermore, even though the extracted content of hallucinogenic substances was 10 times smaller compared to the experiments performed with synthetic psilocin, this confirmed the strong synergistic interaction of psychotropic components of used fungal mushrooms. 

### 2.2. Behavioral Effect—Head-Twitch Response (HTR)

The first stage of the behavioral experiment consisted of the examination of the HTR effect, according to the applied dose of used mushroom extracts (I-II) and psilocin (III). The experiments of this nature give the possibility to determine the inducing of the tested hallucinogenic factors on the serotonergic system. The *head-twitch* response can be defined as the rhythmic side-to-side rotational head movements that occur in mice after the administration of serotonergic hallucinogens and others 5-HT_2A_ agonists [25,26]. The HTR tests are widely used in behavioral assay for studies of 5-HT_2A_ activation and hallucinogen-like effects. The indication of the most effective amounts of hallucinogenic agents administrated in mice can be applied for the investigation of their influence on an activity of serotonin precursor (5-hydroxytryptophan). 

**Figure 2:**
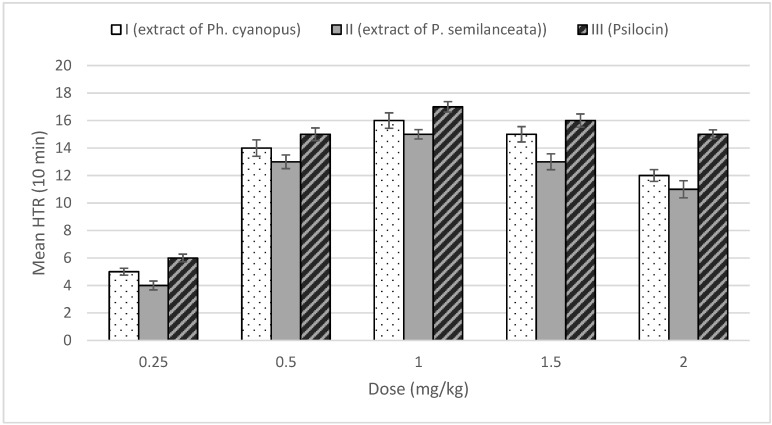
Effect of studied hallucinogenic agents on the head-twitch response in mice, after the administration of the dose. I and II—dried residues of methanolic extracts of *Ph. cyanopus* and *P. semilanceata*, respectively; III—pure psilocin. Data are presented as group means ± S.E.M. using in statistics one-way ANOVA tests with the post hoc tests (Bonferroni procedure).

In the performed experiments, the individuals from the tested population were administered intraperitoneal with doses of fungal extracts and psilocin in the amounts ranged from 0.25 to 2 mg/kg. The number of head twitches was observed after 10 min. The obtained results are illustrated in Figure 2.

It can be seen, that the hallucinogenic components of applied extracts as well as psilocin have been found to elicit head twitch behavior via their actions at 5-HT_2A_ receptors. The binding affinity at 5-HT_2A_ receptors depended on the dose of the used hallucinogenic stimulants (both extracts and pure psilocin). In the control group, only 1–2 spontaneous head twitches were observed. Dose of 1 mg/kg elicited the maximal number of head twitches in mice. Further increasing of the amounts of hallucinogens decreased the number of observed head twitches that was probably due to the regressing saturation of 5-HT_2A_ receptors and their binding to other receptors that might inhibit HTR behavior. The number of head twitches was at its highest when the synthetic psilocin was applied. However, the biological activity of tested fungal extracts displayed a similar behavioral effect (number of HTR) compared to the experiments in which only pure psilocin has been used, despite the fact that the amount of psilocin present in extracts was at much lower concentration level (Table 2). This phenomenon could be related to the synergistic effect of the other indole hallucinogenic alkaloids determined in investigated extracts. This similar effectiveness of the interacting hallucinogenic components of tested extracts with 5-HT_2A_ receptors may be correlated with the occurrence of mannitol (present in the tissues of psychotropic mushrooms) that enables a more efficient transportation of the active substances (hallucinogens) into the brain and thus enhanced their total activity [27].

The stimulation of central serotonin receptors may be observed (as *head-twitch* response) in mice after the administration of the serotonin (5-HT) precursor, 5-hydroxytryptophan (5-HTP)—endogenous full agonist [23,24,25,26,28]. The previous studies demonstrated that the administration of sufficiently large doses of 5-HTP to mice produced spontaneous and irregularly occurring head-twitches [29,30]. The application of a dose larger than 50 mg/kg was required because at this concentration level no HTR effect in mice was observed. In this case, in order to investigate a potency of drug-induced hallucinogenic effect, a dose 200 mg/kg of 5-hydroxytryptophan, administrated after injection of the tested hallucinogenic stimulants was used. 

**Figure 3:**
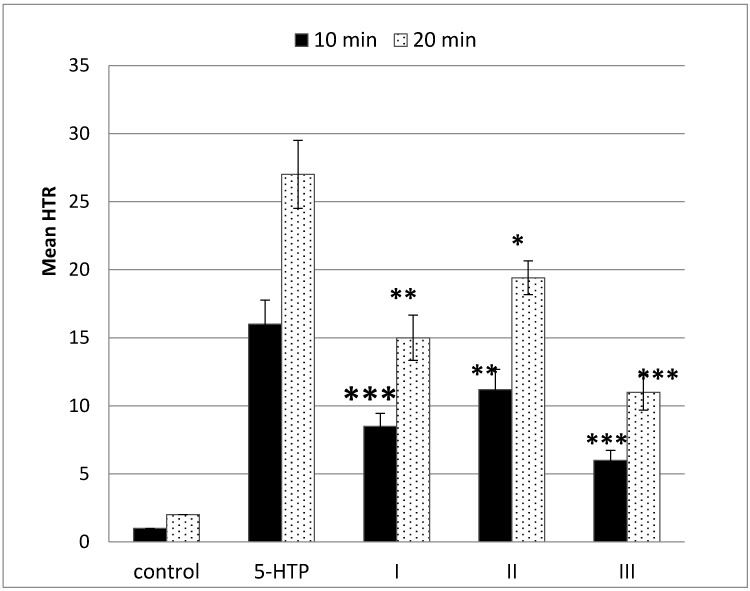
The head-twitch response in mice treated with investigated hallucinogenic stimulants at a dose of 1 mg/kg followed by a single intraperitoneal dose of 200 mg/kg 5-hydroxytryptophan (5-HTP). I—extract of *Ph. cyanopus*; II—extract of *P. semilanceata*; III—pure psilocin. Observation period: 10 and 20 min. Control group was administrated only with isotonic solution of Tween-80. Data are presented as group means ± S.E.M. * *p* < 0.05, ** *p* < 0.01, *** *p* < 0.001 compared to the control group using in statistics one-way ANOVA tests with the post hoc test (Bonferroni procedure).

Figure 3 shows the results of experiments in which tested mushroom extracts and pure psilocin (at dose 1 mg/kg) were injected 15 min before 5-HTP was injected intraperitoneally. The observation period was 10 and 20 min. In control groups of ten mice, only isotonic solution of Tween-80 (1%) was administrated. Tween-80 (a non-toxic and non-ionic additive) was used in order to increase the solubility of dry residues of methanolic mushroom extracts in water. The obtained data indicated that in mice, the tryptamine derivatives (psilocin and active components of applied fungal extracts) produced head-twitches via agonist binding at serotonin 5-HT_2A_ receptors. The used hallucinogenic factors had only partial efficacy at the receptor relative to a full agonist (5-HTP) even at maximal receptor occupancy. The application of pure psilocin gave significantly lower *head-twitch* response (about 60% lower) compared to the effect of full agonist (5-HTP) at dose 200 mg/kg. When a mixture of hallucinogenic substances of fungal extracts was used, the HTR effect (compared to the full agonist activity) was reduced by about 45% (extract of *Ph. cyanopus*) and 30% (extract of *P. semilanceata*). At this point, it should be underlined that the concentration of psilocin in investigated extracts was at much lower level, according to the dose used in experiments with pure psilocin. Furthermore, the observed interactions between the 5-HT_2A_ receptors and hallucinogens of mushroom extracts were also very effective. It seems, that the presence of the other indoleamine hallucinogenic compounds in the used extracts giving the synergistic effect influenced the serotonergic system. 

## 3. Experimental Section 

### 3.1. Mushroom Material

Fruiting bodies of wild mushroom species were collected in the region of southern, south-western and north-eastern Poland in the years 2012 and 2013. The collections of *P. semilanceata* and *Ph. cyanopus* were divided into six representative samples used for further extraction and chromatographic analysis. Mushroom samples were dried (at 40 °C, for 24 h), pulverized and extracted with methanol (Sigma-Aldrich, Poznań, Poland) using ultrasound-assisted process. In this method 500 mg of each mushroom specimen was ground to a powder in a mortar, transferred to a Erlenmeyer flask and, after the addition of 50 mL methanol, placed in an ultrasonic water bath (Cole-Parmer 8891, Vernon Hills, IL, USA) for a period of 3 h, filtered and evaporated to dryness (evaporator: Heidolph, Instruments GmbH&Co. KG, Schwabach, Germany).

### 3.2. LC-MS Analysis

The chromatographic experiments were performed using the UPLC system which consisted of Dionex Ultimate 3000 series including a binary pump, a diode-array detector, an autosampler and a column compartment (Thermo Scientific, San Jose, CA, USA). Methanolic extracts of *P. semilanceata* and *Ph. cyanopus* were separated on a Phenomenex Gemini C18 column (3 μL, 150 × 3.0 mm I.D.; Phenomenex, Torrance, CA, USA) maintained at 35°C. The mobile phase consisted of a mixture 0.2% formic acid (POCH S.A., Gliwice, Poland) in water and a mixture 0.2% formic acid in acetonitrile (Sigma-Aldrich, Poznań, Poland). A constant flow of 0.2 mL/min was applied. The acetonitrile percentages were: 0–1.5 min, 5%; 1.5–12 min, linearly from 5% to 95%; 12–20 min, 95%; 20–25 min, linearly from 95% to 5%; 25–30 min, (equilibration step), 5%. The effluent from the chromatographic column was injected into microOTOFQ-II time of flight mass spectrometer (Bruker Daltonics, Bremen, Germany) equipped with an electrospray ionization (ESI) interface in the positive mode. Mass data were collected in the product ion scan mode. All solvents were of LC-MS grade.

### 3.3. Experimental Animals

The animals were obtained from the breeding facility of the Odessa State Medical University (Odessa, Ukraine). Female outbred mice, weighing between 18 and 24 g were used and were kept in groups of ten during the experimental periods. For the acute toxicity experiments, the total number of mice was 230. In the behavioral test, 150 mice were used to investigate the administrated dose and 50 mice in tests concerning a potency of drug-induced hallucinogenic effect. All animals received a standard laboratory diet and water *ad libitum* and were kept under a continuous 12 h light-dark cycle at room temperature. 

### 3.4. Experimental Procedure

Experimental protocols were approved by the Ethics Committee of the Pharmacological Committee of Ukraine and carried out in strict accordance with the Ethics Committee regulations for the use of experimental animals. The study was conducted in compliance with the Helsinki Declaration for ethical principles in medical research. All efforts were made to minimize animal suffering and the number of animals used. Psilocin (Sigma-Aldrich, Poznań, Poland) and studied hallucinogenic extracts were dissolved in isotonic solution of 1% Tween-80 (Sigma-Aldrich, Poznań, Poland) and administrated intraperitoneally (IP) in a volume of 10 mL/kg. Tween-80 (a non-toxic and non-ionic additive) was used in order to increase the solubility of dry residues of methanolic mushroom extracts in water.

### 3.5. Acute Toxicity Assessment

For the evaluation of acute toxicity, the LD_50_ value was determined. The tested hallucinogenic agents were administrated intraperitoneally in graduated doses to several groups of experimental animals, one dose being used per group. The total number of tested animals in these experiments was 230. The mice received various doses of the substance and after 24 h the number of dead animals was evaluated. The applied range of doses was 200–450 mg/kg for both investigated fungal extracts and 180–420 mg/kg for psilocin. During the study, the behavior of experimental animals affected by the substances was recorded.

### 3.6. Behavioral Effect Assessment

The *head-twitch* responses were used to evaluate a behavioral assay for 5-HT_2A_ activation and to probe for interaction between the 5-HT_2A_ receptor and studied hallucinogenic agents. In order to assess the “effect-dose” dependence, the fungal extracts and psilocin were tested in concentrations ranging from 0.25 to 2 mg/kg. The total number of mice used to study the effect of applied hallucinogenic stimulants on the *head-twitch* response in mice, according to the administrated dose, was 150. 

For the investigation of potency of drug-induced hallucinogenic effects, the dose 200 mg/kg of 5-hydroxytryptophan (Sigma-Aldrich, Poznań, Poland), administrated after the injection of the tested hallucinogenic stimulants, was used. The total number of tested animals in these experiments was 50 mice.

### 3.7. Statistical Methods

The results are expressed as mean ± S.E.M. Data of LD_50_ and HTR were analyzed by using the Student’s *t* test and one-way analysis of variance (ANOVA) followed by the post hoc test (Bonferroni procedure) for multiple comparison. A probability level of *p* < 0.05 was considered significant. The LD_50_ values were calculated by means of the Kerber’s method [31], whereas the substance acute toxicity was evaluated by the Litchfield and Wilcoxon method.

## 4. Conclusions

The studies the acute toxicity of fungal extracts containing psilocin and other indole alkaloids showed similar biological activity to that which was observed when pure psilocin was applied in mice. It should be underlined that the observed toxic effect of mushroom extracts was much stronger compared to pure psilocin effect, especially that the extracts content of hallucinogenic substances was ten times smaller compared to the experiments performed with synthetic psilocin, that confirmed the strong synergistic interaction of psychotropic components of used fungal mushrooms. The comparison of the obtained LD_50_ values for synthetic psilocin and fungal extracts in a Student’s t-test showed insignificant differences (*p* < 0.001). Furthermore, the behavioral test that considered the *head-twitch* response (HTR) was used to assess the effect of the studied psychotropic factors on the serotonergic system. Both, the fungal extracts and psilocin evoked characteristic serotoninergic effect depending on the dose administered to mice, acting as agonist/partial agonist on serotonergic system. The observed behavior of the tested psychotropic agents indicates a high therapeutic potential and the possibility of using this type of drugs for treatment options in mood and anxiety disorders. Clinical literature on serotonergic hallucinogen-like agents has already demonstrated that mushrooms containing psilocybin evoke several beneficial effects, such as a significant decrease in anxiety, improved mood, positive changes in relationships with others and in attitudes in the group of healthy patients and with the advanced-stage of cancer [32,33,34,35]. There is no doubt that the careful preparation and conduction of well-controlled studies in this area will contribute to the increased knowledge of the influence of hallucinogenic substances on the functioning of the central nervous system.
